# The Concentration of C(*sp*^3^) Atoms and Properties of an Activated Carbon with over 3000 m^2^/g BET Surface Area

**DOI:** 10.3390/nano11051324

**Published:** 2021-05-17

**Authors:** Yury M. Shulga, Eugene N. Kabachkov, Vitaly I. Korepanov, Igor I. Khodos, Dmitry Y. Kovalev, Alexandr V. Melezhik, Aleksei G. Tkachev, Gennady L. Gutsev

**Affiliations:** 1 Institute of Problems of Chemical Physics, Russian Academy of Sciences, 142432 Chernogolovka, Russia; en.kabachkov@gmail.com; 2 Institute of New Materials and Nanotechnologies, National University of Science and Technology MISIS, Leninsky pr. 4, 119049 Moscow, Russia; 3 Chernogolovka Scientific Center, Russian Academy of Sciences, 142432 Chernogolovka, Russia; 4 Institute of Microelectronics Technology and High Purity Materials, Russian Academy of Sciences, 142432 Chernogolovka, Russia; korepanov@iptm.ru (V.I.K.); khodos.igor@mail.ru (I.I.K.); 5 Merzhanov Institute of Structural Macrokinetics and Materials Science “ISMAN”, Russian Academy of Sciences, 142432 Chernogolovka, Russia; kovalev@ism.ac.ru; 6 Institute of Technology, Tambov State Technical University, ul. Leningrad 1, 392000 Tambov, Russia; nanocarbon@rambler.ru (A.V.M.); nanotam@yandex.ru (A.G.T.); 7 Department of Physics, Florida A&M University, Tallahassee, FL 32307, USA

**Keywords:** activated carbon with high specific surface area, concentration of *sp*^3^ carbon atoms, Raman spectroscopy, X-ray photoelectron spectroscopy, electron energy-loss spectroscopy

## Abstract

The alkaline activation of a carbonized graphene oxide/dextrin mixture yielded a carbon-based nanoscale material (AC-TR) with a unique highly porous structure. The BET-estimated specific surface area of the material is 3167 m^2^/g, which is higher than the specific surface area of a graphene layer. The material has a density of 0.34 g/cm^3^ and electrical resistivity of 0.25 Ω·cm and its properties were studied using the elemental analysis, transmission electron microscopy (TEM), electron diffraction (ED), X-ray diffraction (XRD), Raman spectroscopy, X-ray photoelectron spectroscopy (XPS), X-ray induced Auger electron spectroscopy (XAES), and electron energy loss spectroscopy (EELS) in the plasmon excitation range. From these data, we derive an integral understanding of the structure of this material. The concentration of *sp*^3^ carbon atoms was found to be relatively low with an absolute value that depends on the measurement method. It was shown that there is no graphite-like (002) peak in the electron and X-ray diffraction pattern. The characteristic size of a *sp*^2^-domain in the basal plane estimated from the Raman spectra was 7 nm. It was also found that plasmon peaks in the EELS spectrum of AC-TR are downshifted compared to those of graphite.

## 1. Introduction

Activated carbons (AC) with high specific surface areas (SSA) are very important for a wide range of industrial applications where they are used as adsorbents for solvents, vapors and pollutants [1,2,3,4]. Their electric conductivity makes them highly promising electrodes for supercapacitors in energy storage applications [5,6,7], and as fillers for thermally conductive polymer composites [8,9,10].

Among a wide variety of high-SSA activated carbons, there is a special class of materials whose SSA measured by the Brunauer-Emmett-Teller (BET) method exceeds the SSA of double-sided graphene sheets (2620–2675 m^2^/g [11,12,13,14,15,16]), which indicates the presence of high concentrations of nanoscale pores and other structure inhomogeneities. One of the basic assumptions of the BET approach is the uniformity of the surface, which is obviously violated for such materials. Nonetheless, one can expect that that the SSA determined using the BET approach is still a useful parameter for characterizing ultraporous carbon materials [17,18].

A common approach used for the production of super-graphene high-porous materials is the alkaline activation of some carbon precursor, typically graphene oxide. For example, the material with *SSA* ~ 3100 m^2^/g was obtained [11] from microwave-exfoliated graphene oxide (MEGO). The chemical activation with KOH is based on the following reaction [19]:

6KOH + 2C = 2K + 3H_2_ + 2K_2_CO_3_ (1)

Alkaline activation has been extensively used to produce AC with SSA > 3000 m^2^/g as shown in Table 1.

Oxidative activation can also be used for this purpose. For example, carbon fiber obtained from pitch was subjected to oxidative activation [32]. One of the interesting findings was that the Raman scattering had a high sensitivity to the precursor material and a very low sensitivity to its SSA, which varied in the range from 1000 to 3000 m^2^/g. It should be noted that even higher SSA values can be found in non-carbonaceous materials. In particular, an SSA value exceeding 5000 m^2^/g was obtained [33] for the polymer derived from zinc-mediated coordination copolymerization of a dicarboxylic and tricarboxylic acid based on the data on low-temperature (77 K) adsorption of N_2_. Metal-organic frameworks (MOFs) are made by linking inorganic and organic units. The surface area values of such MOFs range from 1000 m^2^/g to 10,000 m^2^/g, thus exceeding those of traditional porous materials, such as carbon materials [38]. Recent advancements, technological issues, advantages and drawbacks related to natural gas storage in carbon-based materials and metal−organic frameworks are summarized in [39]. A theoretical substantiation of such high SSA values is based on rolling a spherical probe of a particular size over the atoms of the molecular structure under study. This approach has been applied for the characterization of various materials, and a good agreement between the calculated and BET-measured SSA values was reported [40,41,42,43,44]. This agreement is especially surprising for ultramicroporous materials [44], for which the BET theory assumptions are not fulfilled. Within the framework of this approach, the theoretical SSA maximum is about 15,000 m^2^/g for both crystalline and disordered structures [45]. It is believed that the SSA values measured by the BET method, which are larger than 3000 m^2^/g, reflect the presence of a large number of micropores and/or mesopores in a carbon material.

This work is aimed at a systematic study of the structure of an AC material obtained via an efficient industry-level approach from a graphene oxide/dextrin precursor with carbonization and alkaline activation (the abbreviation for this material is AC-TR). The material has SSA well above 3000 m^2^/g and is conductive. The key parameters of its structure are the content of different carbon fragments, the characteristic size of the *sp*^2^ domains, the stacking mode (or inter-layer structure) of the graphene-like layers and the (de)localization of the electronic excitations inside the material.

Experimental techniques that allow for obtaining these data are TEM, ED, XRD, Raman spectroscopy, XPS, XAES and EELS in the plasmon excitation range. The XPS and EELS techniques were previously used to estimate the *sp*^3^/*sp*^2^ fragment ratio in AC [11,46]. The authors of the study of the MEGO-derived AC material concluded that 98% of all carbon atoms belong to the *sp*^2^ phase [11]. The conclusion on the presence of the *sp*^2^ phase content was based on the EELS study of the C1*s* transitions 1*s* → π * and 1*s* → σ * observed in the range 280–320 eV. The motivation of the present work was to systematically study the structure of the AC-TR material including reliable estimations of the *sp*^3^ phase content by various techniques. Such information is important for engineering 3D graphene-based materials [47,48].

In this work, the concentration of *sp*^3^ states in AC-TR with SSA = 3167 m^2^/g was estimated using numerous techniques. Some methods of characterization of this class of AC materials were used for the first time. It should be noted that the methods used are well known and the results obtained do not depend on the specific surface area of the samples under study. It seems interesting to compare the results obtained by different methods for the same sample with SSA > 3000 m^2^/g, which was not done previously, but can be useful for understanding the structure of such materials.

## 2. Experimental Section

### 2.1. Materials

AC-TR was obtained by alkaline activation at 750 °C of a carbonized at 300 °C mixture of graphene oxide and corn dextrin with the 1:20 mass ratio. After being cooled, the reaction mixture was washed on a filter to remove the alkali. Nest, the mixture was treated with hydrochloric acid to dissolve impurities and washed with water to neutral pH. The resulting material was dried at 110 °C to a constant weight. The AC-TR yield was 30% of the mass of the mixture carbonized at 300 °C. The modified Hummers method was used for GO synthesis [49], and the details of the syntheses are described elsewhere [50,51]. An energy dispersive analysis showed that in addition to carbon, potassium (0.3–0.4 mass %) and oxygen (3–5 mass %) were present in the material. The resistivity and specific gravity of a mixture of AC-TR powders with 15% PTFE were measured on a sample obtained by compressing the mixture at a pressure of 10 MPa in a glass tube with an inner cross-section of 0.06 cm^2^ between steel dies. The specific gravity of the cylinder obtained was 0.33–0.35 g/cm^3^, and the specific electrical resistance was 0.25 Ω·cm.

### 2.2. Equipment Details

The specific surface area for the AC-TR sample was measured with a QUADRASORB *SI* Analyzer (Quantachrome Instruments, Boynton Beach, FL, United States). N_2_ sorption isotherms were obtained at 77 K.

Elemental analysis of AC-TR samples, preliminarily degassed in an argon flow at a temperature of 110 °C for 30 min, was carried out on a CHNS analyzer Vario Micro cube (Elementar GmbH, Hanau, Germany).

Raman spectra were measured with a Bruker Senterra (Billerica, MA, United States) micro-Raman system. The excitation wavelength was 532 nm, the laser power was ~1 mW at the sample point with the beam waist of ~1 µm. Five peaks were used to describe the spectra in the 800–2100 cm^−1^ region: two Gaussians and three pseudo-Vogt functions, as suggested previously [52].

XPS and REELS spectra were obtained using a Specs PHOIBOS 150 MCD9 electron spectrometer (Specs, Berlin, Germany). The vacuum in the spectrometer chamber did not exceed 4 × 10^−8^ Pa. The XPS spectra were excited with an Mg K_α_ radiation (*hν* = 1253.6 eV) and recorded in the constant transmission energy mode (40 eV for the survey spectra and 10 eV for individual lines). The survey spectrum was recorded in 1.00 eV increments, while the spectra of individual lines were recorded in 0.03 eV increments. Background subtraction was carried out according to the Shirley method [53], and the spectra decomposition was performed according to the set of mixed Gaussian/Lorentz peaks in the framework of the Casa XPS 2.3.23 software. The quantification of atomic content was performed using sensitivity factors provided by the elemental library of CasaXPS. The REELS spectra were excited with an electron beam (SPECS EQ 22/35 Electron Source) (Specs, Berlin, Germany). The energy of the exciting electrons was set at 900 eV.

Transmission electron microscopy studies were performed with a JEOL-2100 microscope at the accelerating voltage of 200 kV (JEOL, Tokyo, Japan).

XRD patterns were collected with a DRON (Bourevestnik, St. Petersburg, Russia) diffractometer with Cu Kα radiation (λ = 1.54187Å) and secondary C (002) monochromator under ambient conditions. The XRD data were recorded in a range from 10 to 60° (2θ) with a step of 0.02° with a counting time of 4 s per a step.

## 3. Results and Discussion

### 3.1. Specific Surface Area

**Figure 1:**
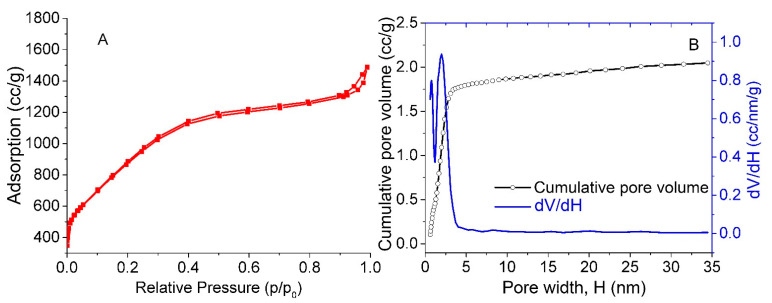
N_2_ adsorption–desorption isotherms (**A**) and pore size distribution curves (**B**) of our sample. The NLDFT method was used to obtain the pore size distribution.

The N_2_ adsorption-desorption isotherms of the sample under study are presented in Figure 1A. The non-localized density functional theory (NLDFT) method was utilized to obtain the pore size distribution shown in Figure 1B, which highlights the presence of both micropores (less than 2 nm) and mesopores (2 nm or more) in the sample. The specific surface area of the sample under study was calculated by the BET method to be 3167 m^2^/g. This value is higher than the previously reported values of activated carbon (1000–2000 m^2^/g) [42,43,44,45]. That is, the presence of numerous pores (2.048 cc/g) contribute to an increase in the surface area. Thus, we are dealing with carbon material with SSA > 3000 m^2^/g in this work.

### 3.2. Elemental Analysis

The data on the composition of the AC-TR sample presented in Table 2 were obtained by elemental analysis by averaging over five experiments. As could be seen, the sum of concentrations of all elements in the first row of the table is less than 100%. Usually, the residue is attributed to oxygen. With this assumption, the sample contains 5.76 wt % oxygen, which presents the estimate from above. The presence of sulfur and nitrogen in the sample is due to their presence in graphene oxide (see, for example, [54]). Note that the presence of elements such as hydrogen and oxygen in the sample may lead to a decrease in the concentration of carbon atoms with *sp*^2^ hybridization due to the chemical bonding of the impurity atoms with the graphene structure.

### 3.3. TEM and ED

**Figure 2:**
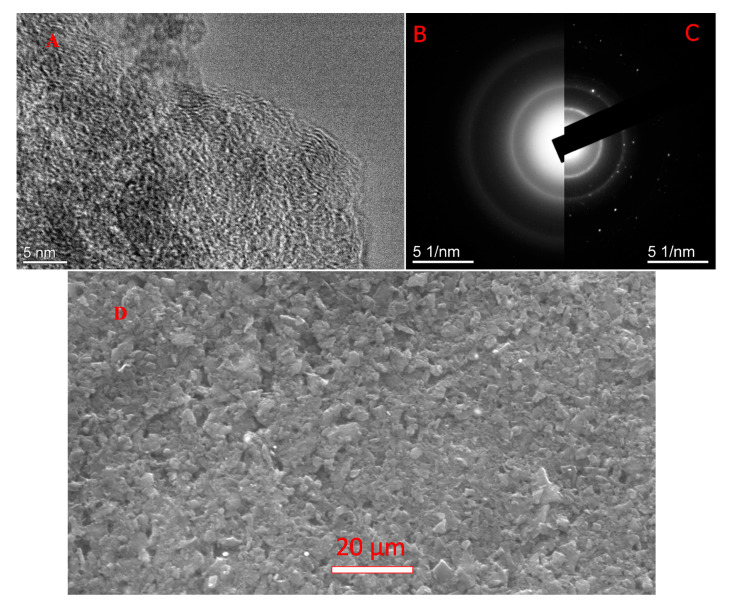
TEM image (**A**) and electron diffraction patterns (**B**) of AC-TR. For comparison, the electron diffraction patterns of polycrystalline graphite (**C**) and SEM image (**D**) are also shown.

Figure 2 shows a TEM image (Figure 2A) and an electron diffraction pattern (Figure 2B) of the AC-TR sample. On the thin edges (Figure 2A), the structure consisting of the crumpled graphene-like layers is clearly resolved. Our TEM image is similar to that obtained for glassy carbon samples pyrolyzed at 600 °C [55]. In the ED pattern (Figure 2B) the first ring belongs to the (100) and (101) reflections. The (002) ring (basal planes reflections), which in polycrystalline graphite (Figure 2C) is located closer to the center of the ED pattern, in AC-TR is either absent or has a minor intensity hidden below a strong halo in the central part of the ED pattern. For comparison, an SEM image is also presented in Figure 2D.

The interplane distance *d* corresponding to the maximum of the first ring of the ED pattern is 0.214 nm. This *d* value for AC-TR is in good agreement with the reference value for graphite: *d_100_* = *a_o_* ($\frac{\sqrt{3}}{2}$) = 0.2131 nm at *a_o_* = 0.2461 nm. It is worth noting that there are no diffraction rings corresponding to diamond (*sp*^3^ bonds) in the ED pattern. The ED results indicate that there is no ABAB stacking, but they do not contradict the stacking itself.

### 3.4. XRD

**Figure 3:**
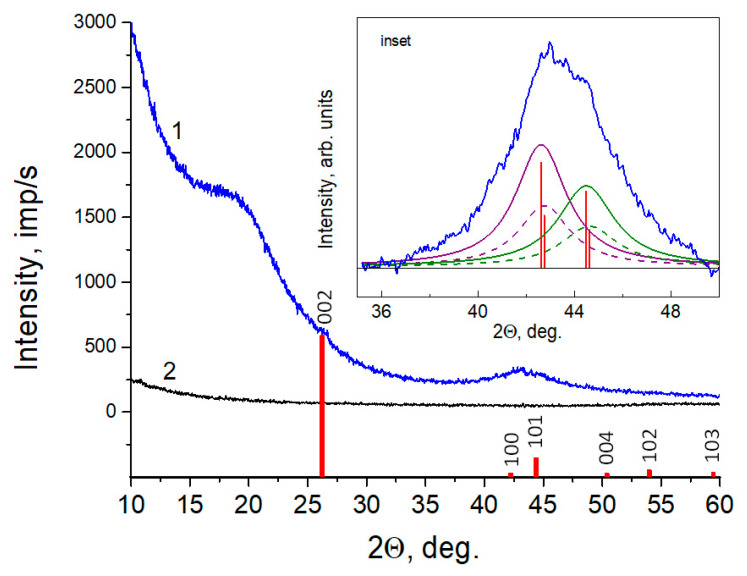
X-ray diffraction patterns of AC-TR powder (1), low background monocrystalline Si plate (2) and stick (red) Carbon PDF2 card#000-75-1621 (http://www.icdd.com (accessed on 2 February 2021)) The inset shows the profile analysis of the diffractogram section.

The absence of the (002) reflection, which is characteristic for *sp*^2^-carbon materials, is also confirmed by X-ray diffraction. Figure 3 shows an XRD pattern of the AC-TR powder, deposited onto a “backgroundless” plate (single crystal Si). For comparison, the graph shows the XRD patterns of the plate before the deposition. According to the patterns, there is no long-range order in the structure of the powder. There is a wide diffuse halo in the region of small angles and a broadened superposition in the range of angles 2θ = 35–55°, which is due to reflections of 100 and 101 from the carbon, belonging to the hexagonal system whose electron diffraction pattern is also presented in Figure 2 for comparison. The profile analysis of the diffraction pattern, considering the splitting of the K_α_ line, makes it possible to separate two reflections. The unit cell parameters of the carbon phase were estimated from the angular position to be a = 2.46 Å and c = 7.34 Å. These values are close to the carbon parameters of the PDF2 card # 000-75-1621 base (a = 2.47Å, c = 6.79Å). The size of the coherent scattering regions estimated by using the Scherrer formula with the integral half-width of (100) reflection, was 2.8 nm. It follows from the analysis of our X-ray and electron diffraction data that the resulting material appears to be structurally close to graphene and is mainly formed by carbon atoms with *sp*^2^ hybridization.

### 3.5. Raman Spectra

**Figure 4:**
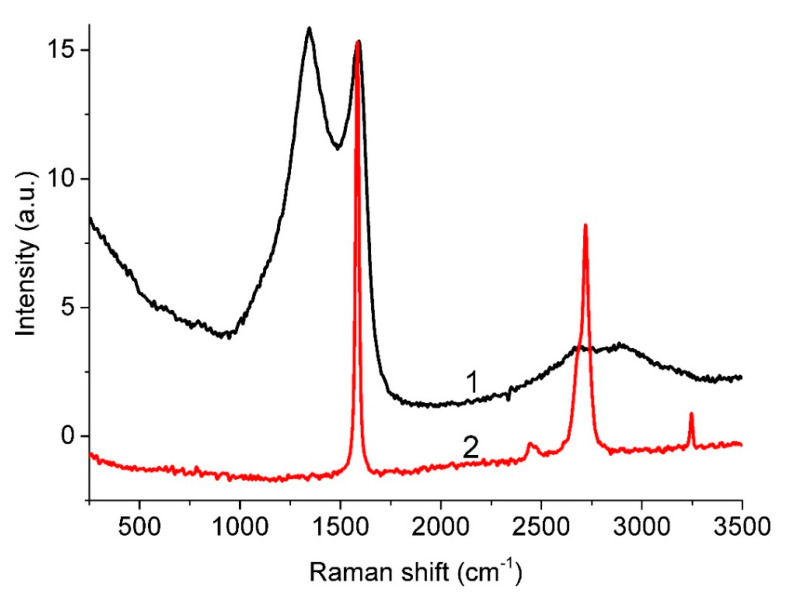
Raman spectra of AC-TR (1) and HOPG (2).

Raman spectroscopy is a powerful technique for the investigation of carbon materials [56] and can be used for the identification of different carbon structures. For example, the vibrational features related to the near-linear arrangement of carbon atoms are usually within the 1800–2200 cm^−1^ spectral region [57,58,59] and are well above the G and D bands (1300–1600 cm^−1^) [60]. Isolated *sp*-carbon species are linear structures with either alternating single and triple bonds (−C≡C−)_n_, called polyenes, or with identical double bonds (=C=C=)_n_, called cumulenes [61]. Zhao et al. reported on the experimental fabrication of linear carbon chains, which were shown to be inside double-walled carbon nanotubes of 0.7 nm in diameter [62]. Three characteristic Raman shift peaks were observed in the range 1790 cm^−1^ to 1860 cm^−1^. The production and characterization of a form of amorphous carbon films with *sp*/*sp*^2^ hybridization (the atomic fraction of *sp* hybridized species was ≥ 20%) has also been reported [63]. In the Raman spectra of these films, the cumulene and polyynes structures corresponded to the peaks at about 1980 cm^−1^ and 2100 cm^−1^, respectively. Figure 4 compares the spectrum of our sample and the spectrum of highly oriented pyrolytic graphite (HOPG). As can be seen, both spectra lack peaks in the range 1800–2200 cm^−1^; therefore, one may conclude that there are no one-dimensional carbon chains in our sample.

Furthermore, it was also reported [56] that, in the Raman spectra excited by 514.5 nm radiation, the cross section of the *sp*^2^ phase is higher, by a factor of 50–250, than the cross section of the *sp*^3^ phase. Consequently, the direct determination of the *sp*^3^/*sp*^2^ ratio from their spectrum is impossible. Since the presence of the *sp*^3^ phase can affect the G peak position, only qualitative estimates can be made.

**Figure 5:**
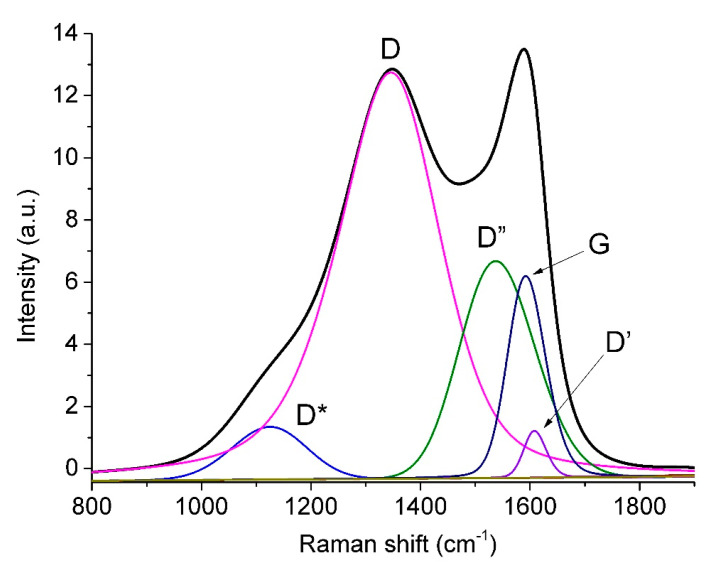
The AC-TR Raman spectrum in the region of 800–1900 cm^−1^ and its five-component deconvolution (bands D *, D, D*”*, G, and D*’*).

Figure 5 presents the fitting of the observed spectrum in the region of 800–1800 cm^−1^ by five functions, whose parameters are given in Table 3. According to the correlation between the positions of peaks D*”* and D* and the oxygen content given in [52,64], the oxygen content in rGO, which presents in our film, is in the range from 0 mass.% (D” position) to 17 mass.% (D* position). A relatively high uncertainty in the oxygen content estimates obtained by this technique is due to the spread of points on the correlation graph [64].

The ratio between the D and G band intensities (*I_D_*/*I_G_*) can serve as a disorder measure in the carbon lattice and can be used [65] for evaluating the size of *sp*^2^-domains *L_a_* as follows:

*L_a_* = (2.4 × 10^−10^) λ^4^_L_ (*I_D_*/*I_G_*)^−^^1^ (2)

where λ_L_ is the wavelength of the exciting laser. It is worth noting that the *L_a_* value for our carbon material evaluated according to Equation (2) equals to 7 nm.

The boundaries of such domains can be considered as *sp*^2^-lattice defects, in particular, they may be formed by *sp*^3^ C atoms located nearby. In the literature, there are various estimates for the C *sp*^3^ concentration obtained from the Raman spectra in different ways. It was noted [66] that the presence of 20% C(*sp*^3^) atoms leads to a decrease in the shift of the G peak from 1600 cm^−1^ to 1510 cm^−1^ in the carbon films with a low content of hydrogen and nitrogen. In our case, the position of the G peak indicates a low fraction of *sp*^3^ carbon atoms.

### 3.6. XPS

**Figure 6:**
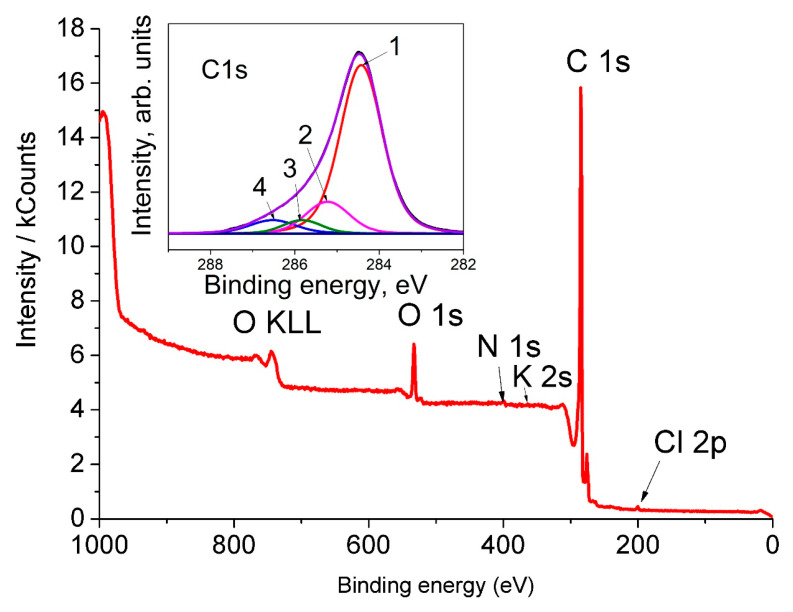
The AC-TR wide XPS spectrum. The inset shows the C1*s* spectrum of the sample and its decomposition (see the text).

The survey XPS spectrum of AC-TR contains the peaks which are mainly due to carbon and oxygen (see Figure 6). The parameters of the C1*s* peak decomposition are presented in Table 2. It can be seen that oxygen in the sample can belong to various functional groups. In addition to an identification of oxygen-containing groups from an analysis of the high-resolution C1*s* spectra, these spectra could be used to determine the *sp*^3^/*sp*^2^ ratio because the energy intervals between the C1*s* peaks corresponding to the C(*sp*^2^) and C(*sp*^3^) states are in the range from 0.4 eV [56] to 0.9 eV [67]. Therefore, to determine the *sp*^3^/*sp*^2^ ratio, one should decompose the C1s peak into two or more peaks. There is no single approach to such a spectrum decomposition and, as a rule, the peak asymmetry is not considered. For example, the intensities of the peaks and the background can be varied, and the positions of the peaks and their half-widths were fixed [56]. Peaks with bond energies of more than 285.1 eV were attributed to carbon atoms, which bond with oxygen [67].

The inset in Figure 6 shows the high-energy resolution C1*s* spectrum of AC-TR after a background subtraction. The results of the C1*s* peak deconvolution performed by using mixed Gauss-Lorentzian functions are presented in Table 4. The assignment of individual peaks was done in accordance with the previous work [68]. It can be seen from Table 4 that the concentration of C(*sp*^3^) in AC-TR is 20%.

### 3.7. XAES

A recent method [68] for estimating the *sp*^3^/*sp*^2^ ratio in carbon materials from their C KVV Auger spectra excited by the X-ray radiation was developed based on the previously proposed method [69]. The method is based on measuring the energy difference between the maximum and the minimum of the first derivative C KVV Auger spectra. This difference, labeled as *D*, can be used for evaluating the *sp*^3^/*sp*^2^ ratio using a linear interpolation of the respective values for diamond (100% of *sp*^3^ bonds) and graphite (100% of *sp*^2^ bonds). The *D* value estimate for C (*sp*^3^) of 13.2 eV can be considered to be reliable. The *D* value for C(*sp*^2^) depends on the oxygen content in a sample. Thus, different *D* values of 23.1 eV and 18.0 eV were determined [68] for HOPG samples with the oxygen content of 0.0 at. % and 7.7 at. %, respectively.

**Figure 7:**
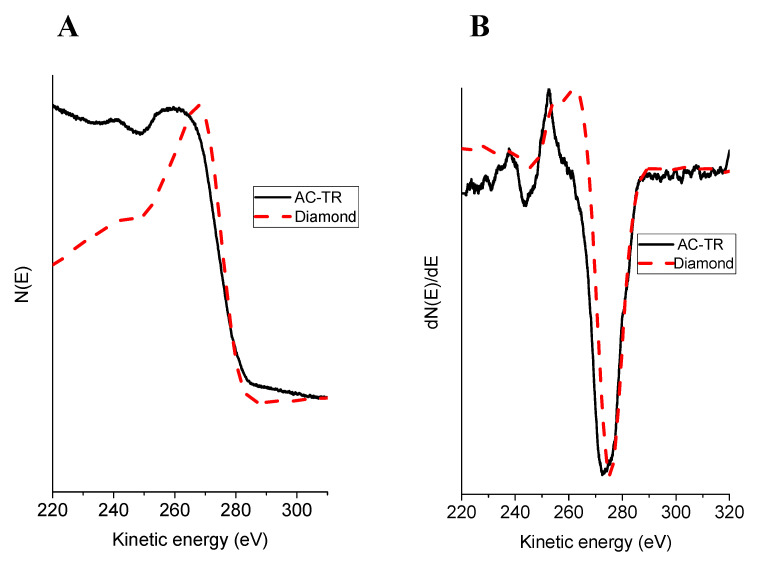
The X-Ray exited Auger C KVV (**A**) and the first derivative C KVV spectra (**B**) for AC-TR and diamond. The spectra in (**A**) were recorded with a step of 0.03 eV; the spectra in (**B**) were obtained by differentiating the corresponding spectra (**A**) using the Savitzky-Golay filter.

Figure 7 displays the measured C KVV Auger spectrum and its first derivative for the sample under study. For comparison, the C KVV spectra of a diamond are shown as the dotted lines. The measured *D* value of 19.6 eV for the AC-TR corresponds to 65% of C(*sp*^2^) when calibrated against the HOPG sample with the oxygen content of 0 at. %. In our sample, the oxygen content according to XPS analysis is 4.69 at. % (see Table 2). Assuming a linear dependence of the slope of the calibration curve on the oxygen content, the estimate for the of C(*sp*^2^) concentration increases to 94%.

### 3.8. Plasmonic REELS

The AC-TR samples with SSA > 3000 m^2^/g were investigated using the EELS technique only in two studies [11,31]. This technique is widely used for the characterization of carbon materials. The most often studied are the losses near the C–K edge, i.e., the losses associated with the excitation from the lowest occupied level of the carbon atom to its lowest vacant level. Practical aspects of the quantification of *sp*^2^-hybridized carbon atoms in the case of diamond-like carbon by electron energy loss spectroscopy are described in [70] and the EELS spectra for carbon materials with different *sp*^3^/*sp*^2^ ratios can be found in recently published papers [55,71,72]. Note that to quantitatively determine the *sp*^3^/*sp*^2^ ratio, it is necessary to separate the loss spectrum from the usually high background (losses due to the multiple scattering). Background subtraction can be performed in different ways that makes quantitative estimates somewhat arbitrary. The magnitude of errors arising from background subtraction in the abovementioned works was not indicated.

After background subtraction, the method of ‘two-window intensity-ratio’ is commonly used for the assessments of the *sp*^3^/*sp*^2^ ratio [73]. It is believed that the ΔEπ and ΔEσ windows are entirely due to the 1s → π* and 1s → σ* transitions, respectively, and the main problem is the choice of the energy window width. There is no single approach for such a choice that makes one expect that the error in determining the *sp*^3^/*sp*^2^ ratio using this method is large. In a study [74] where a more accurate method of fitting was used for separating the π* and σ* parts of the C–K edge spectrum, it was found that the error related with the fitting parameters exceeds 10%. One can anticipate that the error is also larger than 10% if the simpler two-window method is used. Other common methods for determining the *sp*^3^/*sp*^2^ ratio from a carbon-K edge spectrum were considered in [75]. A method was proposed, which was supposed to provide good results on a wide range of samples and should be superior to routine extraction of *sp*^2^ fractions of carbon materials. This method was used [76] in combination with multi-wavelength Raman spectroscopy to study the transformations that occur during the annealing of a C–H film. This work demonstrated the importance of combining several techniques for extracting reliable information on hybridization of carbon atoms. Changes in the shape of the carbon–K edge spectrum of graphene oxide during heating in the range from 70 °C to 1200 °C was analyzed in [77]. It was found that the *sp*^2^ fraction of carbon atoms did not even exceed 85% in the sample annealed at 1200 °C. The results of evaluating the C(*sp*^3^) content in AC with SSA > 3000 m^2^/g (2%, see [11]) are presented above. In [31], the contents of C(*sp*^3^) for two samples of AC with SSA > 3000 m^2^/g estimated by this method were 5% and 6%, respectively.

**Figure 8:**
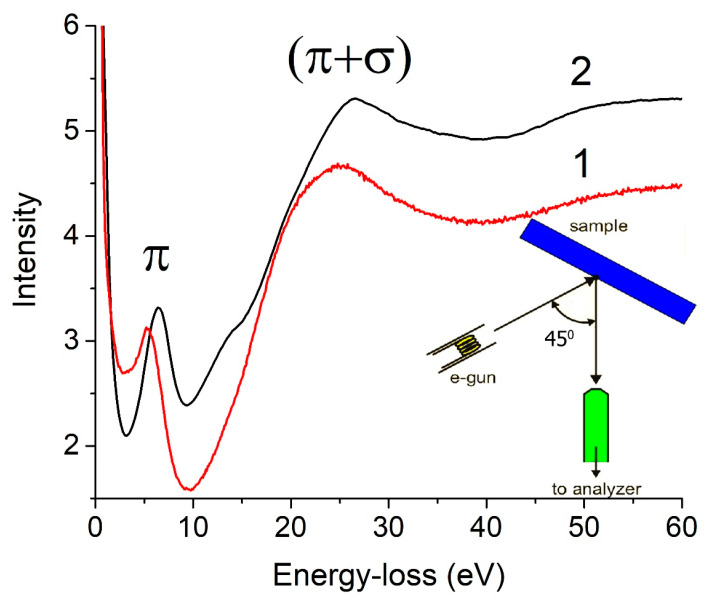
The electron energy-loss spectra of the AC-TR sample (1) and graphite (2) in the reflection geometry. The energy of exciting electrons is 900 eV.

Figure 8 shows the reflection EELS spectra for the sample under study and graphite near the elastic peak. The spectra were normalized to the elastic peak intensity and were not further processed. The peak assignment was carried out according to the literature data [78,79,80,81,82,83,84,85,86] and the peak positions on the energy-loss scale are given in Table 5.

As is known [87], the EELS spectrum of single-layer graphene has two maxima at 4.7 eV (π-plasmon) and 14.5 eV (σ + π-plasmon). With an increase in the number of layers, the maxima move towards higher energies. The limitation of this movement is obvious and corresponds to the position of the plasmon peaks in graphite (see Table 5). Based on these concepts, one can assert that the valence electrons of several graphene layers take part in the plasma oscillations in the sample under study. However, their density is less than that of valence electrons in graphite.

Indeed, the frequency of plasma oscillations (*ω*_p_) of all valence electrons in a solid can be calculated according to the following equation [88]:

*ω*_p_ = (4 *πne*^2^/*mκ*_core_)^1/2^ (3)

where *n* is the density of valence electrons and *κ*_core_ is a parameter related to the polarizability of internal electrons. For carbon materials, the value of *κ*_core_ can be assumed to be unity (for example, *κ*_core_ = 1.0018 for graphite [79]) and, therefore, *n* ~ (*ħω*_p_)^2^. A decrease in the density of valence electrons is possibly associated with an increase in the interlayer distance of the material under study, compared to that in graphite.

In principle, the ratio of the π-plasmon and (σ + π)-plasmon intensities (*I_π_*/*I_σ + π_*) can be used to estimate the effective number of electrons per carbon atom (Ñ_π_), according to the following formula:

Ñ_π_ = *k*(*I_π_*/*I_σ + π_*) (4)

The *k* value was found with an assumption that, for graphite, Ñ_π_ = 1. The application of Equation (4) is complicated by the absence of generally accepted rules for background subtraction. In the present work, we used background subtraction according to [53] when calculating the integral intensities of plasmons. From the data in Table 5, it is easy to find that Ñ_π_ = 0.93 for the sample under study. Note that this value is valid to those spatial regions of the sample where plasma oscillations are localized. Indeed, the energy of 25.0 eV of the main plasmon in the sample under study does not correspond to its low specific density *ρ* = 0.34 g/cm^3^. Therefore, under the assumption

*ħω*_σ + π_~k(*ρ*)^1/2^ (5)

the value *ħω*_σ + π_ = 10.3 eV, when calibrated against that of graphite, whose *ρ* = 2.25 g/cm^3^. This value may be compared to that of fullerite C_60_ whose *ρ* = 1.65 g/cm^3^ and the measured *ħω*_σ + π_ value is 25 eV [89]. One could expect that plasma oscillations are localized on the fullerene-like pore walls in the sample under study.

## 4. Conclusions

Our detailed study of the AC-TR structure has shown that this material can be attributed to the *sp*^2^ class of materials with some inclusion of out-of-plane *sp*^2^-hybridized carbon atoms. The C(*sp*^3^) percentage in the AC-TR material obtained by using of the plasmonic REELS and XAES techniques is 6–7%, which is in good agreement with that of [31] and slightly exceed the estimate made in [11]. Our value of the C(*sp*^3^) content in 20%, based on the XPS measurements, seems to be overestimated because symmetric fitting functions appear to not provide a proper description of the C1*s* spectrum shape. To take into account this asymmetry, it is necessary to use the Doniach-Sunjic functions [90]. According to an analysis based on the Raman spectroscopy data, the content of C(*sp*^3^) atoms is significantly lower than 20%. The presence of hydrogen and oxygen atoms bonded to carbon atoms inside the graphene domains leads to the transitions from C(*sp*^2^) to C(*sp*^3^) atoms. This is a key mechanism for the formation of the *sp*^3^ hybridization of carbon atoms in the AC with SSA exceeding 3000 m^2^/g.

One of the most interesting results of the present work is that there is nographite-like (002) reflection in the diffraction patterns of AC-TR, which typically is the most intense for the *sp*^2^ carbon materials. This means that our sample consists mainly of small single-layered differently folded carbon sheets and does not contain multilayer graphene. According to the data obtained from our measured Raman spectra, the average size of graphene domains is about 7 nm. The plasmon peaks were found to be shifted toward lower energies compared to those of graphite, but these shifts are significantly smaller than could be expected on the basis of the measured specific density of the sample. This can be related to the substantial localization of the plasmonic oscillations.

In conclusion, we note that the electronic and spatial structure of such a promising material as highly porous activated carbon with SSA > 3000 m^2^/g and a sufficiently high electrical conductivity was not yet studied in detail. The results of this study of a representative of this class of materials carried out by using various experimental methods allow one to assert that the content of *sp*^3^ carbon atoms in this *sp*^2^ material is less than 10%.

## Figures and Tables

**Table 1:** SSA values for some carbon materials.

Material and Fabrication Method	Specific Surface Area, m^2^/g	References
alkaline activation of MEGO	3100	[11]
graphene (calculated data)	2620–2675	[12,13,14,15,16,19]
alkali-activated carbon	From 3026 to 3708	[20,21,22,23,24,25,26,27,28,29,30,31]
oxidative activation of pitch-derived carbon fiber	From 1000 to 3000	[32]
polymer derived from zinc-mediated coordination copolymerization of a dicarboxylic and tricarboxylic acid	>5000	[33]
alkaline activation of a mixture graphene oxide and dextrin (AC-TR) carbonized at 750 °C	3167	This work
AC used in supercapacitors	1000–2000	[34,35,36,37]

**Table 2:** Elemental composition of the AC-RT sample.

Content	C	H	N	S	Cl	O	K
wt % ^a^	92.97 ± 0.03	0.451 ± 0.069	0.65 ± 0.07	0.164 ± 0.026			
at % ^b^	94.6 5		0.15	>0.1	0.31	4.69	>0.2

^a^ in weight percent; determined by elemental analysis. ^b^ in atomic percent; determined from X-ray photoelectron spectrum.

**Table 3:** The position (Pos), full width at half maximum (FWHM), and intensity (Int) of each peak in the Raman spectrum of AC-TR.

Peak	Pos, cm^−1^	FWHM, cm^−1^	Int, %
D *	1125.2	160.0	4.9
D	1346.0	220.0	63.1
D*”*	1537.5	161.0	20.3
G	1592.0	81.8	10.3
D*’*	1608.0	48.0	1.4

**Table 4:** The positions (E_b_), intensities (I) and peak assignments in the C1*s* spectrum of AC-TR.

Peak	E_b_, eV	I, %	Assignment
1	284.4	75.87	C (*sp*^2^)
2	285.1	15.23	C (*sp*^3^)
3	285.9	4.38	C (*sp*^3^)–OH
4	286.5	4.51	C (*sp*^2^) = O

**Table 5:** The peak position in the REELS spectra in Figure 7 and the ratio *I_π_/I_σ + π_* of integral intensities.

Sample	π, eV	(σ + π), eV	*I_π_/I_σ + π_*
AC-TR	5.2	25.0	0.134
graphite	6.5	26.5	0.145

## Data Availability

Data available in a publicly accessible repository.
